# Effectiveness of Acceptance and Commitment Therapy (ACT) for the Management of Postsurgical Pain: A Randomized Controlled Trial (SPINE‐ACT Study)

**DOI:** 10.1002/ejp.70293

**Published:** 2026-05-11

**Authors:** Juan R. Castaño‐Asins, Juan P. Sanabria‐Mazo, Jaime Navarrete, Luis M. Martín‐López, Gemma Vila‐Canet, Anna Isart‐Torruella, Alejandro Del Arco‐Churruca, Jesús Lafuente‐Baraza, Gemma Parramon‐Puig, Francina Fonseca‐Casals, Juan V. Luciano, Víctor Pérez‐Solà, Antonio Montes

**Affiliations:** ^1^ Department of Psychiatry, ISM Hospital del Mar Barcelona Spain; ^2^ Department of Psychiatry and Forensic Medicine Autonomous University of Barcelona Cerdanyola del Valles Spain; ^3^ Unit for the Study and Treatment of Pain—ALGOS, Department of Psychology, Research Center for Behavior Assessment (CRAMC) Universitat Rovira i Virgili Tarragona Spain; ^4^ Institut D'investigació Sanitària Pere Virgili Universitat Rovira i Virgili Tarragona Spain; ^5^ Teaching, Research & Innovation Unit, Parc Sanitari Sant Joan de Déu St. Boi de Llobregat Spain; ^6^ Centre of Biomedical Research in Epidemiology and Public Health (CIBERESP) Madrid Spain; ^7^ Spinal Surgery Unit Hospital del Mar Barcelona Spain; ^8^ Department of Mental Health Hospital Universitari Vall D'hebron Barcelona Spain; ^9^ Group of Psychiatry, Mental Health and Addictions Vall D'hebron Research Institute (VHIR) Barcelona Spain; ^10^ Hospital del Mar Research Institute Barcelona Spain; ^11^ Medicine and Life Sciences Department Niversitat Pompeu Fabra Barcelona Spain; ^12^ Department of Clinical & Health Psychology Autonomous University of Barcelona Cerdanyola del Valles Spain; ^13^ Centre of Biomedical Research in Mental Health (CIBERSAM) Madrid Spain; ^14^ Pain Clinic Unit, Anesthesiology Hospital del Mar Barcelona Spain

## Abstract

**Background:**

This randomized controlled trial evaluated the effectiveness of group‐based online Acceptance and Commitment Therapy (ACT) compared with treatment as usual (TAU) in improving postsurgical outcomes in patients with degenerative lumbar conditions.

**Methods:**

A total of 91 participants scheduled for lumbar surgery were randomly assigned to either ACT or TAU, with primary analyses conducted in a modified intention‐to‐treat sample of participants who underwent surgery (*n* = 54). Outcomes and process variables were assessed at baseline, post‐treatment (pre‐surgery), and at 3‐, 6‐ and 12‐month post‐surgery follow‐up.

**Results:**

Linear mixed model analysis showed that ACT (vs. TAU) produced larger reductions in pain interference (primary outcome) at post‐treatment (*d* = 1.86) and at 3‐month (*d* = 1.82), 6‐month (*d* = 1.49) and 12‐month post‐surgery follow‐up (*d* = −1.68). ACT yielded greater improvements than TAU in secondary outcomes, including depressive/anxiety symptoms (*d* = 1.69–1.99), pain catastrophizing (*d* = 0.84–0.97), kinesiophobia (*d* = 0.76–0.92) and low back pain‐related disability (*d* = 1.39–1.91); and process variables, including psychological flexibility (*d* = 1.71–2.44) and pain acceptance (*d* = 2.33–3.04). Across time, no significant between‐group differences in pain severity were observed. No indirect effects of ACT (vs. TAU) on outcomes through process variables were found. Clinically relevant number needed to treat values for pain interference response were obtained at 3‐month (NNT = 4), 6‐month (NNT = 2) and 12‐month (NNT = 4) post‐surgery follow‐ups.

**Conclusions:**

Overall, these findings support the importance of delivering ACT before surgery to improve pain‐related outcomes.

**Significance Statement:**

This randomized controlled trial provides evidence that preoperative group‐based Acceptance and Commitment Therapy (ACT) improves pain‐related outcomes following lumbar surgery. ACT produced reductions in pain interference, depressive/anxiety symptoms, pain catastrophizing, kinesiophobia, low back pain‐related disability, psychological flexibility and pain acceptance, with clinically meaningful numbers needed to treat. These findings extend existing evidence by demonstrating the effectiveness of ACT delivered before surgery and support the integration of perioperative psychological interventions into routine surgical care for patients with degenerative lumbar conditions.

## 1 Introduction

Chronic low back pain (CLBP) is a complex condition in which biopsychosocial factors shape its clinical presentation (Gallach‐Solano et al. 2022). Degenerative lumbar conditions are commonly a cause of CLBP and are associated with reduced psychological function (Abbas et al. 2013). Surgical treatment for lumbar degenerative conditions has demonstrated benefits over non‐surgical approaches (Kreiner et al. 2013), yet up to 40% of patients report pain intensity, disability, and poor quality of life after surgery (Weinstein et al. 2010). Reoperation rates range from 18% to 23% within 8–10 years (Lurie et al. 2015), further underscoring the need for adjunctive strategies to improve long‐term outcomes beyond those achieved with surgery alone.

Prospective studies have identified risk factors for postoperative pain, including psychological factors associated with pain perception and functional outcomes (Sobol‐Kwapinska et al. 2016). Evidence suggests that high preoperative levels of depression, anxiety, kinesiophobia, and pain catastrophizing are related to greater pain intensity and disability after surgery (Giusti et al. 2021). These findings point to modifiable psychosocial targets for intervention in the perioperative period (Nadinda et al. 2022), supporting psychological therapies aimed at reducing negative affect, avoidance behaviours, and pain perception after surgery (Castaño‐Asins et al. 2025; Nadinda et al. 2022).

Randomized clinical trials (RCTs) have shown that perioperative psychological therapies based on Acceptance and Commitment Therapy (ACT) and mindfulness‐based interventions (MBIs) are effective in managing postoperative pain and disability (Castaño‐Asins et al. 2025; Nadinda et al. 2022). Specifically, ACT has robust evidence of effectiveness in improving pain interference, psychological flexibility, and quality of life among people with chronic pain (Lai et al. 2023; Ma et al. 2023; Martinez‐Calderon et al. 2024).

ACT encourages individuals to change their relationship with feared or avoided thoughts and physical sensations through processes such as acceptance, mindfulness, and value‐based committed action (Hayes et al. 2014). Research has shown that psychological flexibility and pain acceptance are key processes linked to improvements in pain‐related outcomes (Navarrete et al. 2025; Trompetter et al. 2015). These findings suggest that enhancing psychological flexibility and pain acceptance in the perioperative period may be clinically relevant; however, evidence on the effectiveness of preoperative ACT in surgical populations remains limited.

This RCT aimed to evaluate the effectiveness of preoperative group‐based ACT, delivered in addition to treatment‐as‐usual (TAU), in improving pain interference (primary outcome), pain intensity, anxiety/depressive symptoms, functional status, kinesiophobia, and pain catastrophizing (secondary outcomes) and psychological flexibility and pain acceptance (process variables) in patients with scheduled surgery for degenerative lumbar pathology; and to examine the role of psychological flexibility and pain acceptance as potential mediators of change of long‐term changes in pain interference. Based on previous research, we hypothesize that (1) ACT would yield greater improvements across primary and secondary outcomes compared to TAU alone (Castaño‐Asins et al. 2025; Nadinda et al. 2022; Veehof et al. 2016) and (2) improvements in primary and secondary outcomes would be mediated by increases in psychological flexibility and pain acceptance within the ACT group (Lin et al. 2018; Sanabria‐Mazo et al. 2023; Trompetter et al. 2015).

## 2 Methods

### 2.1 Study Design

A 12‐month, single‐blinded RCT was conducted with patients randomly allocated to two treatment arms: (1) ACT + TAU (hereafter, ACT) and (2) TAU alone (hereafter, TAU). Assessments were conducted at baseline, post‐treatment (2 months after baseline and before surgery), and at 3‐, 6‐ and 12‐month post‐surgery follow‐up. The RCT was registered at ClinicalTrials.gov (NCT05634122) and followed the Consolidated Standards of Reporting Trials (CONSORT) (Hopewell et al. 2025).

The study was conducted in accordance with the Declaration of Helsinki and was approved by the Institutional Ethics Committee of Hospital del Mar (2021; Reference 2021/9998/I). Patients or members of the public were not involved in the design, conduct, or reporting of this study. However, patient and public involvement is planned to ensure the dissemination of the main results in an accessible and meaningful manner. No financial incentives were provided for study participation. The study protocol has been described in detail elsewhere (Castaño‐Asins et al. 2023).

### 2.2 Sample Size

The trial preregistration (ClinicalTrials.gov NCT05634122) indicated an initial recruitment target of 80 participants, whereas the study protocol (Castaño‐Asins et al. 2023) reported a planned sample size of 102 participants based on 80% statistical power to detect the expected between‐group difference in the primary outcome. The preregistration reflected an initial feasibility‐based estimate, whereas the study protocol was informed by the formal sample size calculation. Recruitment feasibility in this single‐center surgical population was constrained by strict eligibility criteria, the limited pool of surgical candidates, and the need to avoid prolonging the trial beyond the protocol‐defined timeline. Consequently, 91 eligible patients were enrolled and randomized (ACT = 48; TAU = 43). After randomization, 10 participants (5 per arm) were excluded due to post‐randomization eligibility errors (i.e., participants who were randomized but, upon review of clinical records, later found to have prior lumbar surgery), leaving 81 participants with baseline data. This represents the maximum feasible recruitment within the study period. Consistent with CONSORT 2025 (Hopewell et al. 2025), the deviation from the planned sample size is transparently documented here and reflects feasibility constraints.

### 2.3 Participants

**FIGURE 1:**
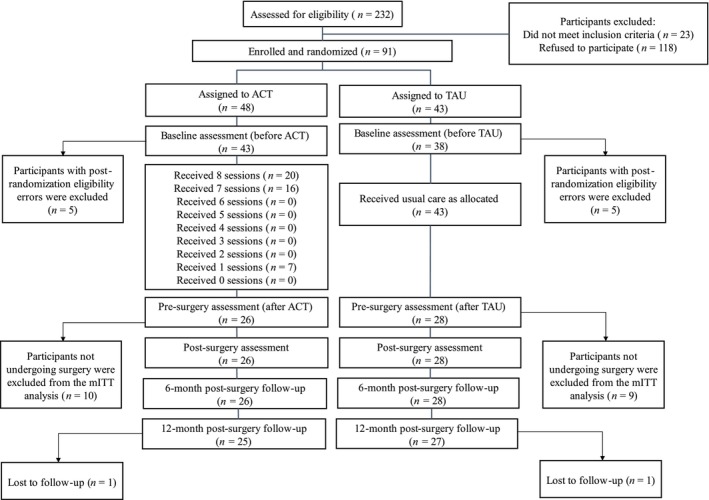
Flowchart of participants in the RCT.

Participants were recruited from the Spine Unit of Hospital del Mar (Barcelona, Spain). Patients scheduled for lumbar surgery due to degenerative low back pathology and presenting psychosocial risk factors for chronic postsurgical pain (CPSP) were consecutively assessed for eligibility and invited to participate in the RCT. Of the 232 patients assessed for eligibility, 91 met the selection criteria and were recruited between February 2022 and October 2024. These participants were randomly allocated to two study arms: ACT (*n* = 48) and TAU alone (*n* = 43). As shown in Figure 1, the modified intention‐to‐treat (mITT) sample comprised 54 participants who completed baseline assessment and finally underwent surgery (ACT, *n* = 26; TAU, *n* = 28).

Inclusion criteria were (a) age 18–80 years; (b) adequate understanding of Spanish; (c) diagnosis of degenerative low back pain indicating first‐time lumbar surgery according to clinical history and International Classification of Diseases diagnostic and surgical codes (ICD‐10), a criterion used to reduce prognostic heterogeneity associated with previous lumbar surgeries (Shologan et al. 2025); (d) presence of psychosocial risk factors for CPSP; and (e) access to an internet connection on a smartphone, tablet, or computer to participate in online therapy. Psychosocial risk factors associated with CPSP were defined as anxiety, depression, pain catastrophizing, and fear of movement (Giusti et al. 2021). Concretely, the indication for first‐time lumbar surgery was considered when patients showed lumbar spinal stenosis, foraminal stenosis, lumbar spondylolisthesis, lumbar degenerative disc disease, degenerative scoliosis, lumbar kyphosis, and a herniated disc. To assess the presence of psychosocial risk factors, patients were required to report at least one psychosocial risk factor associated with a poor postoperative prognosis. Consistent with established cutoffs, patients scoring ≥ 11 on the Hospital Anxiety and Depression Scale (HADS; Zigmond and Snaith 1983), ≥ 24 on the Pain Catastrophizing Scale (PCS; Sullivan et al. 1995), or ≥ 27 on the Tampa Scale of Kinesiophobia (TSK‐11; Eiger et al. 2019) were classified as being at elevated psychosocial risk for chronic postsurgical pain.

Exclusion criteria were (a) cognitive impairment that prevented participation in therapy; (b) presence of chronic pain not related to degenerative low back pain requiring surgical treatment; (c) absence of psychosocial risk factors for CPSP; (d) involvement in litigation or pending legal claims for compensation; (e) severe or decompensated psychiatric disorder (e.g., severe depression, schizophrenia, bipolar disorder, or personality disorder) that prevented participation in therapy; (f) suicidal ideation; (g) substance abuse or dependence (except smoking); and (h) indication for lumbar reoperation.

### 2.4 Procedure

Patients who met the eligibility criteria were informed about the study and were contacted by the first author (JRC‐A) to address any questions. This contact typically occurred within 1–3 weeks after the initial screening. Interested patients were invited to attend a face‐to‐face baseline visit at the hospital's pain clinic. During this visit, the first author explained the study procedures and confidentiality safeguards, obtained written informed consent, and administered the baseline clinical and self‐report assessments. Eligibility was formally confirmed at this visit by reassessing the prespecified psychological risk criteria using standardized instruments. Assessments were conducted in person at the hospital using paper‐and‐pencil questionnaires completed by participants during the visit. Patient data were treated confidentially, with access restricted to the research team after personal identifiers had been recoded. Only the first author had access to the code key, which in accordance with Spanish data protection legislation was stored in a secure location separate from the code.

Randomization occurred after completion of the baseline assessments, in line with CONSORT recommendations (Hopewell et al. 2025). Recruitment was conducted in seven recruitment cohorts. The number of participants included in each cohort was as follows: Cohort 1 (*n* = 21), Cohort 2 (*n* = 10), Cohort 3 (*n* = 9), Cohort 4 (*n* = 12), Cohort 5 (*n* = 9), Cohort 6 (*n* = 20), and Cohort 7 (*n* = 10). At the end of each cohort, a computer‐generated randomization list in SPSS (version 26) was used to allocate participants in a 1:1 ratio to either the control group (TAU) or intervention group (ACT). This procedure ensured that each wave was independently randomized and preserved the integrity of the allocation sequence. Randomization was conducted by a member of the research team who was not involved in patient recruitment or outcome assessment. Allocation concealment was maintained until after completion of the baseline assessment.

### 2.5 Interventions

Patients were informed that their participation was voluntary and that they could withdraw at any time, with the assurance that they would continue to receive TAU. They were also informed that their usual medical management, including pharmacological treatment, would not be altered by participation in the RCT. Patients underwent non‐instrumented procedures, such as laminectomy or foraminoplasty, or instrumented procedures, such as laminectomy with posterior arthrodesis or anterior arthrodesis, depending on clinical indication. Before the start of the trial, the ACT intervention was standardized and delivered by a psychiatrist (JRC‐A) with more than 5 years of clinical experience delivering ACT. The therapist received specific training in the study protocol to ensure treatment fidelity. Brief between‐session homework tasks and reminders were used to support engagement with the therapy. Trial interventions have been described in detail in the published protocol (Castaño‐Asins et al. 2023).

#### 2.5.1 Acceptance and Commitment Therapy (ACT)

ACT is a contextual cognitive‐behavioural intervention that aims to increase psychological flexibility by fostering openness to unwanted experiences, present‐moment awareness, and engagement in values‐based action (Hayes et al. 2014). The ACT program consisted of 8 weekly group sessions of approximately 90 min, delivered via videoconference during the preoperative period before lumbar surgery, with up to 15 participants per group. ACT was based on the Vowles et al. (2008) protocol. An outline of the ACT sessions is detailed in Table 1.

**TABLE 1:** Outline of ACT sessions.

Session	Description
1	Introduction of participants and clinicians, rationale for ACT, and psychoeducation on chronic pain, depression, and stress. Presentation of models of pain and suffering, identification of personal values, and brief breathing/attention exercises
2	Initial work on personal values and patterns of experiential avoidance. Use of metaphors to illustrate how attempts to control internal experiences can be counterproductive. Exploration of anxiety and fight–or–flight responses within the context of one's life journey
3	Further clarification of values and objectives. Discussion of how thoughts and language influence behaviour. Introduction of cognitive defusion strategies to create distance from difficult thoughts, together with basic mindfulness/meditation practices
4	Identification of psychological barriers and obstacles to valued living. Exploration of emotional distress and its consequences, including personality and health‐related factors. Formulation of initial committed actions aligned with values
5	Deepening work on values and emotions. Development of a concrete ‘plan for action and willingness’ for values‐based behaviour. Emphasis on psychological flexibility, resilience, motivation, and body‐focused exercises (e.g., body scan, relaxation)
6	Clarification of life direction and long‐term values. Introduction of self‐as‐context and present‐moment awareness (‘here and now’). Discussion of brain–emotion links and practice of skills for managing intense emotions, assertiveness, and self‐esteem
7	Experiential expansion exercises to increase openness to bodily sensations and internal experiences. Integration of elements from positive psychology (meaning, enjoyment, gratitude) and discussion of the benefits of physical activity and movement
8	Integration of ACT skills learned across sessions. Reflection on personal changes and consolidation of committed actions. Application of ACT strategies to the upcoming spinal surgery and postoperative recovery, followed by group closure and farewell

#### 2.5.2 Treatment‐As‐Usual (TAU)

TAU consisted of medication prescription and routine follow‐up according to standard clinical practice and each patient's symptoms. Usual management included pharmacological treatment and general recommendations regarding physical activity and self‐care. Patients randomized exclusively to TAU did not receive any additional psychological intervention during the study period. After completion of follow‐up assessments, patients in the TAU group were offered the opportunity to receive the ACT program if their healthcare providers deemed it clinically appropriate.

### 2.6 Measures

#### 2.6.1 Sociodemographic and Clinical Data

We gathered data about age, sex, educational level, marital status, and employment status. Clinical data related to pain history and surgical indication included the healthcare provider's diagnosis and the type of surgery indicated for degenerative lumbar pathology.

#### 2.6.2 Primary Outcome

The Brief Pain Inventory (BPI) was used to measure pain interference (primary outcome) and pain intensity (secondary outcome) during the past week (Badia et al. 2003; Daut et al. 1983). The BPI is an 11‐item self‐report questionnaire, with four items assessing pain intensity over the last 24 h on a 0–10 scale (0 = ‘No pain’, 10 = ‘Pain as bad as you can imagine’), and seven items assessing pain interference with general activity, mood, walking, work, relations with others, sleep, and enjoyment of life on a 0–10 scale (0 = ‘Does not interfere’, 10 = ‘Completely interferes’). Higher scores indicate greater pain intensity and greater pain interference. Internal consistency in this study was good across time points for pain interference (Cronbach's alpha [*α*] = 0.89–0.99).

#### 2.6.3 Secondary Outcomes

The HADS (Bjelland et al. 2002; Herrero et al. 2003) was used to assess anxiety/depressive symptoms during the past week. The HADS is a 14‐item self‐report questionnaire comprising items of anxiety and depressive symptoms. Items are rated on a 4‐point Likert scale from 0 (‘Not at all’) to 3 (‘All the time’), and a total score of emotional distress can be obtained by summing all the items (range 0–42). Higher scores indicate greater anxiety/depressive symptoms. Internal consistency in this study was good across time points (*α* = 0.94–0.95).

The PCS (García‐Campayo et al. 2008; Sullivan et al. 1995) was used to assess pain catastrophizing. The PCS is a 13‐item self‐report questionnaire comprising three dimensions: rumination, magnification, and helplessness. Items are rated from 0 (‘Not at all’) to 4 (‘All the time’), with a total score from 0 to 52. Higher scores indicate greater pain catastrophizing. Internal consistency of the total score in this study was excellent across time points (*α* = 0.93–0.98).

The TSK (Gómez‐Pérez et al. 2011; Swinkels‐Meewisse et al. 2003) was used to assess pain‐related fear of movement (kinesiophobia). The TSK is an 11‐item self‐report questionnaire rated on a 4‐point Likert scale from 1 (‘Strongly disagree’) to 4 (‘Strongly agree’), with a total score ranging from 11 to 44. Higher scores indicate greater kinesiophobia. Internal consistency in this study was excellent across time points (*α* = 0.90–0.96).

The *Oswestry Low Back Pain Disability Scale* (OLBPDQ; Koivunen et al. 2024) was used to assess low back pain‐related disability. The OLBPDQ is a 13‐item self‐report questionnaire covering pain intensity and limitations in daily activities (self‐care, lifting, walking, sitting, standing, sleeping, sexual activity, social life and travel). Each item has 6 response options, scored from 0 to 5, with higher scores indicating greater limitation. The total score is obtained by summing item scores, dividing by the maximum possible score, and multiplying by 100 to yield a percentage (0%–100%), with higher values indicating greater disability. Score ranges are typically interpreted as 0%–20% (minimal disability), 21%–40% (moderate), 41%–60% (severe), 61%–80% (crippled), and > 80% (bed‐bound or exaggerating symptoms). Internal consistency in this study was good across time points (*α* = 0.82–0.94).

#### 2.6.4 Process Variables

The *Psychological Inflexibility of Pain Scale* (PIPS; Rodero et al. 2013; Wicksell et al. 2010) was used to assess psychological inflexibility related to pain. The PIPS is a 12‐item self‐report questionnaire rated on a 7‐point Likert‐type scale ranging from ‘Never true’ to ‘Always true’. Higher scores indicate greater psychological inflexibility. Internal consistency in this study was good across time points (*α* = 0.87–0.99).

The *Chronic Pain Acceptance Questionnaire* (CPAQ; McCracken et al. 2004; Rodero et al. 2010) was used to assess pain acceptance. The CPAQ is a 20‐item self‐report questionnaire comprising two subscales: activity engagement and pain willingness. Items are rated from 0 (‘Never true’) to 6 (‘Always true’), with a total score ranging from 0 to 120. Higher scores indicate greater pain acceptance. Internal consistency in this study was good across time points (*α* = 0.81–0.93).

### 2.7 Statistical Analyses

All analyses were computed using SPSS (v26) and Mplus (v7.4). Descriptive statistics were presented as means and standard deviations for continuous variables and as frequencies and percentages for categorical variables. Baseline between‐group differences in sociodemographic and clinical variables were examined using Student's *t* tests or χ^2^ tests. Following CONSORT recommendations and methodological recommendations (Gruijters 2016), baseline sociodemographic and clinical differences after randomization were not included as covariates in the outcome analyses; however, they are reported descriptively.

Linear mixed models (LMM) with restricted maximum likelihood estimation were used to compare the effects of ACT versus TAU on outcomes and process variables at post‐treatment (2 months after baseline and before surgery) and at 3‐, 6‐, and 12‐month post‐surgery follow‐up, using a modified intention‐to‐treat (mITT) approach (Abraha and Montedori 2010). The post‐treatment assessment was included to evaluate the immediate effects of the preoperative ACT intervention before the potential influence of the surgical procedure on outcomes. The mITT analysis included all randomized participants who underwent surgery and completed baseline assessment. This approach was selected because the intervention targeted perioperative outcomes, and participants who did not undergo surgery were not exposed to the relevant surgical context. Exploratory ITT analyses, including all randomized participants, showed a similar pattern of results; therefore, we focus on the mITT analyses to facilitate interpretation of the main findings. The 6‐month post‐surgery follow‐up was the primary endpoint for evaluating the intervention's effect on the primary outcome, given that 6‐month follow‐up is commonly used as an endpoint in lumbar spine surgery trials (McGregor et al. 2013; Weinstein et al. 2009).

LMM included a random intercept to account for within‐participant correlations over time and fixed effects for group (ACT vs. TAU), time, and the group × time interaction, adjusting for baseline values of the corresponding outcome and time from pre‐surgery assessment to surgery. Treatment effects were estimated from the group × time interaction at each time point and are reported as regression coefficients with 95% confidence intervals. Model assumptions, including normality, homoscedasticity of residuals, and the absence of influential outliers, were assessed using histograms, boxplots, Q–Q plots, residuals scatterplots, and skewness and kurtosis values, with no major violations noted. The planned sensitivity analyses using a per‐protocol approach could not be conducted because the proportion of participants who attended fewer than 6 of the 8 ACT sessions was small. However, a sensitivity analysis additionally adjusted for sex to assess the robustness of estimates. According to recent publications, sex has been associated in the literature with differences in clinical presentation and postoperative outcomes following surgery for degenerative lumbar spine disease. Females are often reported to experience greater pain and disability both preoperatively and postoperatively (MacLean et al. 2024). Effect sizes (Cohen's *d*) were calculated for each group comparison using pooled baseline standard deviations to standardize pre–post and pre–follow‐up mean differences. A two‐tailed significance level of 0.05 was used in all tests. To control for multiple comparisons across outcomes and time points, the Benjamini–Hochberg procedure was applied to the set of *p*‐values to control the false discovery rate at *α* = 0.05.

To determine the clinical significance of changes in the primary outcome, participants were classified as responders or non‐responders based on the Initiative on Methods, Measurement and Pain Assessment in Clinical Trials (IMMPACT; Dworkin et al. 2005) recommendations. A reduction of ≥ 1 point from baseline in BPI interference at the 6‐month follow‐up was considered the primary criterion for clinically important improvement, with the same threshold also examined at the 3‐ and 12‐month follow‐ups. This classification was also used to calculate the number needed to treat (NNT) and the absolute risk reduction (ARR) for ACT versus TAU, with 95% confidence intervals. Baseline comparisons of sociodemographic and clinical variables between responders and non‐responders, as planned in the RCT protocol, were not conducted due to the low proportion of non‐responders.

Mediation analyses were conducted to examine whether changes in psychological flexibility (PIPS) and pain acceptance (CPAQ) mediated the effects of ACT on the treatment outcomes. A series of simple mediation analyses was tested, in which the independent variable was always the treatment condition (TAU = 0, ACT = 1), the mediator variables were the change scores on CPAQ or PIPS, and the dependent variables were the change scores on the primary and secondary outcomes. Change scores were calculated for CPAQ and PIPS from baseline to post‐treatment and for the treatment outcomes from baseline to 6‐month follow‐up (clinical endpoint). Post hoc mediation analyses using change scores in the primary and secondary outcomes from baseline to the 3‐month and 12‐month follow‐up assessments were also conducted for exploratory purposes. Path analyses were conducted, modelling the direct effect of the independent variable both on the mediator (*a*) and dependent (*c*) variable, as well as the direct effect of the mediator on the dependent variable (*b*). In addition, the indirect (*a* × *b*) effect of the independent variable on the dependent variable through the mediator was estimated using bias‐corrected bootstrap procedures with 10,000 resamples. Mediation effects were considered statistically significant when the 95% confidence interval did not include zero. To control for multiple testing, the Benjamini–Hochberg procedure was applied to the set of indirect effect *p*‐values to control the false discovery rate at *α* = 0.05.

## 3 Results

### 3.1 Participant Flow and Compliance

Of the 232 patients assessed for eligibility, 23 did not meet the inclusion criteria, and 118 declined to participate. A total of 91 participants were randomized to this RCT, with 48 assigned to ACT and 43 to TAU; however, 10 participants (5 per study arm) were excluded after randomization because a subsequent review of clinical records identified prior lumbar surgery, which violated the prespecified inclusion criterion of first‐time lumbar surgery. Baseline data were therefore available for 43 participants in the ACT group and 38 in the TAU group. The mean number of sessions attended by participants in the ACT group was 6.5 (SD = 2.5). As shown in Figure 1, 36 (84%) participants assigned to ACT completed 7 or 8 sessions. Overall, 27 of the 81 participants with baseline data dropped out of the study. The main reasons for dropout were logistic or personal reasons without undergoing surgery (*n* = 8), the decision to pursue conservative treatment (*n* = 4), medical reevaluation indicating that surgery was no longer indicated (*n* = 5), voluntary refusal of surgery due to fear or family concerns (*n* = 7), and serious medical complications (*n* = 3).

Participants who ultimately underwent surgery comprised the mITT sample (26 in the ACT group and 28 in the TAU group), with no additional dropouts at pre‐surgery, post‐surgery, 3‐month, or 6‐month follow‐up assessments. At the 12‐month follow‐up, one participant in the ACT group had not yet reached the assessment time point, and one participant in the TAU group had died. There were no statistically significant differences (*p* > 0.05) in baseline sociodemographic, clinical, or psychological characteristics between participants included in the mITT analysis (*n* = 54) and those not included (*n* = 37).

### 3.2 Sociodemographic and Clinical Characteristics

Most participants were middle‐aged adults with primary or secondary education. Approximately half were females, most lived with a partner, and the majority were either working or retired at study entry. Participants presented with long‐standing low back pain, most commonly due to lumbar spinal stenosis or degenerative disc disease. Pain was typically localized to a single site. Most participants were scheduled to undergo a laminectomy with or without posterior arthrodesis. The mean interval between the pre‐surgery assessment and surgery was 120 days (SD = 116.11; ranging from 1 to 460). In total, 13.2% of participants presented with a single psychological risk factor, whereas the majority (86.8%) presented with two or more concurrent factors. Anxiety–depressive disorders were the most common psychiatric comorbidities. The mean duration of back pain was about 7 years. More information on baseline sociodemographic, clinical, and psychological characteristics is provided in Table 2.

**TABLE 2:** Baseline characteristics of participants by study arm.

Variables	ACT (*n* = 26)	TAU (*n* = 28)	*p*
Sex (female), *n* (%)	9 (34.6)	14 (50)	0.25
Age, mean (SD)	58.12 (13.05)	60.03 (15.01)	0.61
Marital status, *n* (%)			
Single	3 (11.7)	3 (10.7)	0.89
Married/living with partner	21 (80.7)	23 (82.1)	
Separated/divorced	1 (3.8)	1 (3.6)	
Widowed	1 (3.8)	1 (3.6)	
Living arrangement, *n* (%)			
Living alone	4 (15.6)	6 (17.9)	0.41
Living with a partner	22 (84.4)	22 (82.1)	
Education level, *n* (%)			
Illiterate	1 (3.8)	1 (3.6)	**0.02**
Primary studies	4 (15.4)	15 (53.6)	
Secondary studies	15 (57.7)	11 (39.2)	
University	6 (23.1)	1 (3.6)	
Employment status, *n* (%)			
Homemaker	1 (3.8)	1 (3.6)	0.20
Paid employment	2 (7.7)	8 (28.5)	
Paid employment, but on sick leave	12 (46.2)	7 (25)	
Unemployed with/without subsidy	1 (3.8)	1 (3.6)	
Retired/pensioner	7 (26.9)	4 (14.3)	
Temporal disability	3 (11.5)	7 (25)	
Back pain diagnosis, *n* (%)			
Lumbar spinal stenosis	10 (38.5)	10 (35.7)	0.07
Foraminal stenosis	1 (3.8)	0 (0)	
Lumbar spondylolisthesis	5 (19.2)	8 (28.6)	
Degenerative disc disease	10 (38.5)	4 (14.3)	
Degenerative scoliosis	0 (0)	2 (7.1)	
Disc herniation	0 (0)	4 (14.3)	
Surgical procedure, *n* (%)			
Laminectomy	10 (38.5)	8 (28.6)	0.45
Foraminoplasty	1 (3.8)	0 (0)	
Laminectomy with posterior arthrodesis	10 (38.5)	16 (57.1)	
Anterior arthrodesis	5 (19.2)	4 (14.3)	
Pain location, *n* (%)			0.05
Single	26 (100)	24 (85.7)	
Multiple	0 (0)	4 (14.3)	
Comorbid mental disorders, *n* (%)			
Anxious‐depressive adjustment disorder	25 (96.2)	26 (92.8)	0.62
Major depressive disorder, single episode	1 (3.8)	1 (3.6)	
Generalized anxiety disorder	0 (0)	1 (3.6)	
Clinical variables			
Years of back pain diagnosis, *M* (SD)	7.88 (6.99)	6.57 (4.68)	0.42
Days from pre‐surgery assessment to surgery, *M* (SD)	132.54 (103.76)	100.86 (94.85)	0.25
Outcomes, *M* (SD)			
BPI interference (0–10)	7.71 (1.11)	8.26 (1.14)	0.08
BPI intensity (0–10)	6.04 (1.66)	6.61 (1.09)	0.14
HADS‐T (0–42)	21.46 (5.96)	17.71 (6.99)	**0.04**
PCS (0–52)	33.58 (4.37)	31.29 (6.56)	0.14
PIPS (12–84)	60.65 (7.45)	61.75 (6.26)	0.56
TSK‐11SV (11–44)	29.92 (6.80)	32 (7.14)	0.28
CPAQ‐20 (0–120)	32.69 (9.31)	34.96 (11.09)	0.42
OLBPDQ (0–100)	30.46 (4.24)	29.07 (4.89)	0.27

*Note:* Significant differences in bold (*p* < 0.05).

Abbreviations: ACT, Acceptance and Commitment Therapy; BPI‐I, Brief Pain Inventory‐Interference; BPI‐S, Brief Pain Inventory‐Severity; CPAQ, Chronic Pain Acceptance Questionnaire; HADS‐T, Hospital Anxiety and Depression Scale‐Total score; OLBPDQ, Oswestry Low Back Pain Disability Scale; PCS, Pain Catastrophizing Scale; PIPS, Psychological Inflexibility of Pain Scale; TAU, Treatment as usual; TSK, Tampa Scale of Kinesiophobia.

### 3.3 Effects on Pain Interference (Primary Outcome)

**FIGURE 2:**
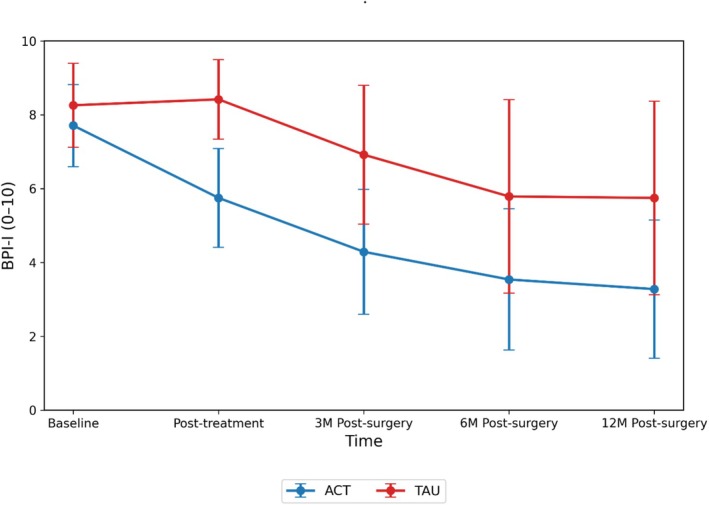
Pain interference (BPI‐I) over time in the ACT and TAU groups.

Table 3 presents between‐group analyses for pain interference (BPI‐I) using the mITT approach. After applying the Benjamini–Hochberg correction for multiple comparisons, ACT produced significantly greater reductions in pain interference than TAU at post‐treatment (*β* = −2.12, *p* < 0.001), 3‐month post‐surgery follow‐up (*β* = −2.08, *p* < 0.001), 6‐month post‐surgery follow‐up (*β* = −1.70, *p* < 0.001), and 12‐month post‐surgery follow‐up (*β* = −1.95, *p* < 0.001). Figure 2 shows the BPI‐I scores over time in the ACT and TAU groups.

**TABLE 3:** Linear mixed model results for primary and secondary outcomes (mITT approach).

	ACT (*n* = 26)	TAU (*n* = 28)	ACT vs. TAU		
	*M* (SD)	*M* (SD)	*d*	*t* (*p*)	*β* (95% CI)
**Primary outcome**					
BPI‐I [0–10]					
Baseline	7.71 (1.11)	8.26 (1.14)			
Post‐treatment	5.75 (1.34)	8.42 (1.08)	−1.86	**−4.53 (< 0.001)**	−2.12 [−3.05, −1.20]
Post‐surgery	4.29 (1.69)	6.92 (1.88)	−1.82	**−4.44 (< 0.001)**	−2.08 [−3.00, −1.16]
6‐month follow‐up	3.54 (1.91)	5.79 (2.62)	−1.49	**−3.63 (< 0.001)**	−1.70 [−2.63, −0.78]
12‐month follow‐up	3.28 (1.87)	5.75 (2.62)	−1.68	**−4.09 (< 0.001)**	−1.95 [−2.89, −1.01]
**Secondary outcomes**					
BPI‐S [0–10]					
Baseline	6.04 (1.66)	6.61 (1.09)			
Post‐treatment	6.34 (1.57)	6.89 (0.89)	0.01	0.03 (0.980)	0.01 [−0.96, 0.99]
Post‐surgery	4.26 (1.66)	5.24 (1.75)	−0.29	−0.84 (0.404)	−0.41 [−1.39, 0.56]
6‐month follow‐up	3.09 (2.27)	4.43 (2.44)	−0.55	−1.47 (0.119)	−0.77 [−1.75, 0.20]
12‐month follow‐up	2.83 (2.25)	4.37 (2.45)	−0.69	−1.72 (0.088)	−0.88 [−1.88, 0.13]
HADS‐T [0–42]					
Baseline	21.46 (5.96)	17.71 (6.99)			
Post‐treatment	11.65 (3.83)	21.04 (7.51)	−1.99	**−9.42 (< 0.001)**	−13.13 [−15.88, −10.37]
Post‐surgery	10.58 (4.83)	18.39 (8.50)	−1.75	**−8.29 (< 0.001)**	−11.56 [−14.32, −8.81]
6‐month follow‐up	9.42 (4.40)	16.82 (9.51)	−1.69	**−7.99 (< 0.001)**	−11.15 [−13.90, −8.39]
12‐month follow‐up	9.4 (4.05)	16.56 (9.15)	−1.65	**−7.73 (< 0.001)**	−10.78 [−13.53, −8.03]
PCS [0–52]					
Baseline	33.58 (19.05)	31.29 (6.56)			
Post‐treatment	22.96 (6.57)	32.68 (7.31)	−0.84	**−5.76 (< 0.001)**	−12.01 [−16.13, −7.89]
Post‐surgery	18.62 (8.03)	28.25 (9.85)	−0.84	**−5.72 (< 0.001)**	−11.93 [−16.05, −7.81]
6‐month follow‐up	15.54 (8.10)	27 (11.31)	−0.97	**−6.60 (< 0.001)**	−13.75 [−17.87, −9.63]
12‐month follow‐up	14.76 (8.04)	26.15 (11.17)	−0.96	**−6.53 (< 0.001)**	−13.60 [−17.71, −9.50]
TSK‐11SV [11–44]					
Baseline	29.92 (6.80)	32 (7.14)			
Post‐treatment	24.31 (6.93)	32.89 (6.99)	−0.92	**−4.23 (< 0.001)**	−6.51 [−9.55, −3.47]
Post‐surgery	20.81 (6.11)	28.50 (7.97)	−0.79	**−3.65 (< 0.001)**	−5.61 [−8.65, −2.58]
6‐month follow‐up	18.58 (5.93)	26.04 (7.70)	−0.76	**−3.50 (< 0.001)**	−5.38 [−8.42, −2.34]
12‐month follow‐up	19.20 (6.02)	26 (7.14)	−0.67	**−3.37 (< 0.001)**	−5.12 [−8.11, −2.12]
OLBPDQ [0–100]					
Baseline	60.92 (8.47)	58.14 (9.78)			
Post‐treatment	51.54 (11.42)	62.36 (9.17)	−1.46	**−3.60 (< 0.001)**	−13.60 [−21.07, −6.13]
Post‐surgery	42.77 (13.26)	54 (13.80)	−1.51	**−3.71 (< 0.001)**	−14.01 [−21.48, −6.54]
6‐month follow‐up	33.54 (15.54)	48.57 (17.85)	−1.91	**−4.71 (< 0.001)**	−17.81 [−25.28, −10.34]
12‐month follow‐up	26.40 (11.06)	36.52 (13.05)	−1.39	**−3.70 (< 0.001)**	−13.23 [−20.28, −6.19]
**Process variables**					
PIPS [12–84]					
Baseline	60.65 (7.45)	61.75 (6.26)			
Post‐treatment	44.58 (6.77)	62.64 (6.73)	−2.44	**−6.06 (< 0.001)**	−16.97 [−22.50, −11.44]
Post‐surgery	40.50 (7.97)	54.14 (11.29)	−1.80	**−4.48 (< 0.001)**	−12.55 [−18.08, −7.01]
6‐month follow‐up	36.81 (9.05)	49.82 (15.29)	−1.71	**−4.25 (< 0.001)**	−11.92 [−17.45, −6.38]
12‐month follow‐up	36.04 (9.96)	49.85 (15.55)	−1.83	**−4.52 (< 0.001)**	−12.82 [−18.41, −7.23]
CPAQ‐20 [0–120]					
Baseline	32.69 (9.31)	34.96 (11.09)			
Post‐treatment	61.38 (11.54)	32 (9.84)	3.04	**10.31 (< 0.001)**	31.66 [25.59, 37.72]
Post‐surgery	64.23 (11.65)	42.21 (12.56)	2.33	**7.91 (< 0.001)**	24.29 [18.22, 30.36]
6‐month follow‐up	66.38 (6.42)	42.79 (14.68)	2.48	**8.42 (< 0.001)**	25.87 [19.80, 31.94]
12‐month follow‐up	67.68 (6.54)	44.74 (12.29)	2.42	**8.87 (< 0.001)**	25.85 [20.10, 31.59]

*Note:* Means and standard deviations are based on raw (unadjusted) data. At the 12‐month follow‐up, two participants, one in each group, were lost to follow‐up (ACT, *n* = 25; TAU, *n* = 27). Values in brackets indicate the possible score range for each questionnaire. All estimates correspond to the group × time interaction effects derived from linear mixed models. The variable time from pre‐surgery assessment to surgery was not statistically significant in any comparison. After applying the Benjamini–Hochberg procedure, the tests with *p* < 0.001 remained statistically significant (adjusted *p* = 0.001), whereas the remaining tests (original *p* = 0.119, 0.404, and 0.980) were not significant. In the sensitivity analyses, sex was not statistically significant in any comparison (all *p* ≥ 0.05). Significant values (*p* < 0.05) are shown in bold.

Abbreviations: ACT, Acceptance and Commitment Therapy; B, regression coefficients; BPI‐I, Brief Pain Inventory‐Interference; BPI‐S, Brief Pain Inventory‐ Severity; CI, confidence interval; CPAQ, Chronic Pain Acceptance Questionnaire; d, Cohen's *d* as an effect size measure; HADS‐T, Hospital Anxiety and Depression Scale‐Total score; M, mean; mITT, modified intention‐to‐treat; OLBPDQ, Oswestry Low Back Pain Disability Scale; PCS, Pain Catastrophizing Scale; PIPS, Psychological Inflexibility of Pain Scale; SD, standard deviation; TAU, Treatment as‐usual; TSK, Tampa Scale of Kinesiophobia.

### 3.4 Effects on Pain Intensity, Depressive/Anxiety Symptoms, Pain Catastrophizing, Kinesiophobia, and Low Back Pain‐Related Disability (Secondary Outcomes)

Between‐group analyses for pain severity (BPI‐S), depressive/anxiety symptoms (HADS‐T), pain catastrophizing (PCS), kinesiophobia (TSK‐11SV), and low back pain‐related disability (OLBPDQ) are shown in Table 3 according to the mITT approach. After applying the Benjamini–Hochberg correction, no significant between‐group differences in pain severity were found at any assessment point. In contrast, ACT produced significantly greater reductions in depressive/anxiety symptoms than TAU at post‐treatment (*β* = −13.13, *p* < 0.001), 3‐month post‐surgery follow‐up (*β* = −11.56, *p* < 0.001), 6‐month post‐surgery follow‐up (*β* = −11.15, *p* < 0.001), and 12‐month post‐surgery follow‐up (*β* = −10.78, *p* < 0.001). Likewise, ACT showed larger reductions in pain catastrophizing at post‐treatment (*β* = −12.01, *p* < 0.001), 3‐month post‐surgery follow‐up (*β* = −11.93, *p* < 0.001), 6‐month post‐surgery follow‐up (*β* = −13.75, *p* < 0.001), and 12‐month post‐surgery follow‐up (*β* = −13.60, *p* < 0.001); in kinesiophobia at post‐treatment (*β* = −6.51, *p* < 0.001), 3‐month post‐surgery follow‐up (*β* = −5.61, *p* < 0.001), 6‐month post‐surgery follow‐up (*β* = −5.38, *p* < 0.001), and 12‐month post‐surgery follow‐up (*β* = −5.12, *p* < 0.001); and in low back pain‐related disability at post‐treatment (*β* = −13.60, *p* < 0.001), 3‐month post‐surgery follow‐up (*β* = −14.01, *p* < 0.001), 6‐month post‐surgery follow‐up (*β* = *−*17.81, *p* < 0.001), and 12‐month post‐surgery follow‐up (*β* = −13.23, *p* < 0.001).

### 3.5 Effects on Psychological Flexibility and Pain Acceptance (Process Variables)

Table 3 presents between‐group analyses for psychological flexibility (PIPS) and pain acceptance (CPAQ‐20) using the mITT approach. After applying the Benjamini–Hochberg correction, ACT produced significantly greater reductions in psychological inflexibility than TAU at post‐treatment (*β* = −16.97, *p* < 0.001), 3‐month post‐surgery follow‐up (*β* = −12.55, *p* < 0.001), 6‐month post‐surgery follow‐up (*β* = −11.92, *p* < 0.001), and 12‐month post‐surgery follow‐up (*β* = −12.82, *p* < 0.001). Similarly, ACT yielded significantly larger increases in pain acceptance than TAU at post‐treatment (β = 31.66, *p* < 0.001), at 3‐month post‐surgery follow‐up (*β* = 24.29, *p* < 0.001), at 6‐month post‐surgery follow‐up (*β* = 25.87, *p* < 0.001), and at 12‐month post‐surgery follow‐up (*β* = 25.85, *p* < 0.001).

### 3.6 Number Needed to Treat (NNT)

Responders were defined as participants achieving at least a 1‐point reduction in pain interference (BPI‐I) from baseline. At the 3‐month post‐surgery follow‐up, 24 out of 26 participants in ACT (92.3%) and 18 of 28 in TAU (64.3%) met this criterion; at the 6‐month post‐surgery follow‐up, 25 of 26 participants in ACT (96.2%) and 8 of 28 in TAU (28.6%) were classified as responders; and at the 12‐month post‐surgery follow‐up, 19 of 25 participants in ACT (76%) and 14 of 27 in TAU (51.9%) were classified as responders. At 3 months, the ARR for ACT versus TAU was 28% (95% CI 7.5%–48.5%), corresponding to an NNT of 4 (95% CI 2.3–15.5). At 6 months, the ARR was 67.6% (95% CI 49.3%–85.9%), yielding an NNT of 2 (95% CI 1.2–2.0). At 12 months, the ARR was 24.2% (95% CI −1.1%–49.5%), corresponding to a point estimate NNT of 4; however, because the ARR confidence interval includes zero, this result should be interpreted with caution.

### 3.7 Role of Psychological Flexibility and Pain Acceptance as Treatment Mediators of the Study Outcomes

Table 4 shows the unstandardized regression coefficients (with standard errors in parentheses), 95% confidence intervals, and *p*‐values for the direct (paths *a*, *b* and *c*) and indirect (*a* × *b*) effects of the tested mediation models. None of the models yielded statistically significant indirect effects of study arm on clinical outcomes. Taking the primary outcome as an example, participants in the ACT arm (vs. TAU) showed a significant decrease in PIPS change scores (*a* = −16.97, SE = 1.92, *p* < 0.001), but neither PIPS change scores (*b* = 0.01, SE = 0.05, *p* = 0.792) nor receiving ACT (*c* = −1.49, SE = 1.02, *p* = 0.144) significantly predicted BPI‐I change scores. Accordingly, the indirect effect of the intervention on BPI‐I change scores through PIPS change scores was not statistically significant (*a × b* = −0.21, SE = 0.83, *p* = 0.797). Again, although ACT (vs. TAU) participants showed a significant increase in CPAQ‐20 change scores (*a* = 31.66, SE = 3.34, *p* < 0.001), neither CPAQ‐20 change scores (*b* = −0.02, SE = 0.03, *p* = 0.473) nor receiving ACT (*c* = −1.08, SE = 1.10, *p* = 0.325) significantly predicted BPI‐I change scores. Accordingly, the indirect effect of the intervention on BPI‐I change scores through CPAQ‐20 was also not statistically significant (*a × b* = −0.62, SE = 0.89, *p* = 0.485).

**TABLE 4:** Mediation analyses of ACT (versus TAU) effect on primary (BPI‐I) and secondary (BPI‐S, HADS‐T, PCS, TSK‐11SV, and OLBPDQ) outcomes through PIPS and CPAQ‐20 change scores.

	Path	PIPS			CPAQ‐20		
		Coeff. (SE)	95% CI	*p*	Coeff. (SE)	95% CI	*p*
BPI‐I	*a*	−16.97 (1.92)	[−19.99, −13.62]	**< 0.001**	31.66 (3.34)	[25.88, 36.84]	**< 0.001**
	*b*	0.01 (0.05)	[−0.07, 0.09]	0.792	−0.02 (0.03)	[−0.06, 0.03]	0.473
	*c*	−1.49 (1.02)	[−3.24, 0.08]	0.144	−1.08 (1.10)	[−3.02, 0.58]	0.325
	*a* × *b*	−0.21 (0.83)	[−1.55, 1.13]	0.797	−0.62 (0.89)	[−2.02, 0.89]	0.485
BPI‐S	*a*	−16.97 (1.92)	[−19.99, −13.62]	**< 0.001**	31.66 (3.34)	[25.88, 36.84]	**< 0.001**
	*b*	−0.00 (0.05)	[−0.09, 0.09]	0.949	−0.00 (0.03)	[−0.05, 0.05]	0.883
	*c*	−0.83 (1.16)	[−2.78, 1.03]	0.473	−0.64 (1.19)	[−3.10, 1.22]	0.589
	*a* × *b*	0.06 (0.93)	[−1.47, 1.56]	0.950	−0.13 (0.91)	[−1.85, 1.42]	0.885
HADS‐T	*a*	−16.97 (1.92)	[−19.99, −13.62]	**< 0.001**	31.66 (3.34)	[25.88, 36.84]	**< 0.001**
	*b*	0.20 (0.14)	[−0.03, 0.44]	0.160	−0.02 (0.07)	[−0.14, 0.10]	0.742
	*c*	−7.74 (2.87)	[−12.58, −3.30]	0.007	−10.38 (3.01)	[−15.62, −5.76]	0.001
	*a* × *b*	−3.41 (2.41)	[−7.34, 0.51]	0.157	−0.77 (2.33)	[−4.31, 3.27]	0.743
PCS	*a*	−16.97 (1.92)	[−19.99, −13.62]	**< 0.001**	31.66 (3.34)	[25.88, 36.84]	**< 0.001**
	*b*	0.22 (0.22)	[−0.15, 0.58]	0.313	−0.06 (0.11)	[−0.22, 0.13]	0.570
	*c*	−9.96 (4.27)	[−17.35, −3.39]	0.020	−11.84 (4.33)	[−19.39, −5.30]	0.006
	*a* × *b*	−3.79 (3.88)	[−10.37, 2.26]	0.328	−1.91 (3.42)	[−7.21, 3.98]	0.576
TSK‐11SV	*a*	−16.97 (1.92)	[−19.99, −13.62]	**< 0.001**	31.66 (3.34)	[25.88, 36.84]	**< 0.001**
	*b*	0.12 (0.11)	[−0.06, 0.29]	0.246	−0.06 (0.07)	[−0.17, 0.06]	0.369
	*c*	−3.30 (2.23)	[−6.63, 0.68]	0.139	−3.38 (2.90)	[−8.03, 1.52]	0.244
	*a* × *b*	−2.09 (1.94)	[−5.55, 0.79]	0.282	−2.01 (2.29)	[−5.70, 1.74]	0.381
OLBPDQ	*a*	−16.97 (1.92)	[−19.99, −13.62]	**< 0.001**	31.66 (3.34)	[25.88, 36.84]	**< 0.001**
	*b*	−0.11 (0.43)	[−0.79, 0.61]	0.806	−0.06 (0.27)	[−0.48, 0.38]	0.815
	*c*	−19.62 (8.78)	[−34.02, −5.62]	0.026	−15.85 (10.33)	[−32.80, 1.09]	0.125
	*a* × *b*	1.80 (7.45)	[−10.56, 13.27]	0.809	−1.97 (8.61)	[−16.47, 11.67]	0.819

*Note:* Mediation analyses were computed with completers (i.e., patients who had been assessed in the four evaluation periods); *n* = 54 in all cases. Direct effects are *a*, *b*, and *c* paths. Indirect effects are × *b*. Unstandardized coefficients are provided. Significant values (*p* < 0.05) are shown in bold.

Abbreviations: BPI‐I, Brief Pain Inventory‐Interference; BPI‐S, Brief Pain Inventory‐ Severity; CPAQ, Chronic Pain Acceptance Questionnaire; HADS‐T, Hospital Anxiety and Depression Scale‐Total score; OLBPDQ, Oswestry Low Back Pain Disability Scale; PCS, Pain Catastrophizing Scale; PIPS, Psychological Inflexibility of Pain Scale; TSK, Tampa Scale of Kinesiophobia.

Post hoc mediation analyses, including change scores in the primary and secondary outcomes from baseline to 3‐month follow‐up, yielded the same result, with one exception. There was a significant indirect effect of ACT (vs. TAU) on HADS change scores through PIPS change scores (*a* × *b* = −5.04, SE = 2.10, *p* = 0.016). However, the Benjamini–Hochberg correction for multiple testing indicated that this statistically significant result was spurious. The same result was observed at the 12‐month follow‐up, with no significant indirect effects.

## 4 Discussion

This RCT examined the effectiveness of ACT for improving postoperative pain‐related functioning in patients with degenerative lumbar spine pathology. In addition, we tested whether theoretically relevant process variables mediated the clinical effects of ACT. Compared with TAU, ACT produced significantly greater improvements in pain interference at post‐treatment and the three post‐surgery follow‐ups, as well as in anxiety/depressive symptoms, pain catastrophizing, kinesiophobia, and low back pain‐related disability. Thus, Hypothesis 1 was supported for all outcomes except for pain severity, which decreased similarly over time in both groups, with a non‐significant trend favoring ACT. Overall, these changes were not statistically mediated by changes in pain acceptance or psychological flexibility; therefore, Hypothesis 2 was not supported in this sample.

Our findings are consistent with the broader ACT literature in the chronic pain population. Current evidence shows that ACT enhances pain‐related functioning, pain acceptance, psychological flexibility, and mindfulness, as well as reduces depressive/anxiety symptoms, pain catastrophizing, and psychological inflexibility from post‐treatment through long‐term follow‐up, while effects on pain intensity are smaller and less consistent (Martinez‐Calderon et al. 2024). Moreover, the significant improvement in pain functioning in favour of the ACT group is consistent with previous studies showing that perioperative psychological interventions can modulate patients' metabolic stress response to surgery and, in turn, improve surgical outcomes (Lanini et al. 2022). These findings should, however, be interpreted with caution, given the sample size.

In this line, the improvement in depressive/anxiety symptoms seems quite significant when considering the interplay between psychological factors and perioperative physiological responses, such as wound repair and immunity. Villa et al. (2020) systematically reviewed clinical studies on the effectiveness of brief psychological support for adults having elective abdominal or urologic surgery before or around the time of their surgery. The authors found support for the effectiveness of these interventions on reducing postoperative pain and lower anxiety, with a few also showing reduced need for pain medication. Indeed, poor mental health is a main contributor to chronic opioid use after surgery (Rhon et al. 2018), further highlighting the interest of the present results.

It is noteworthy that participants receiving only TAU showed a similar improvement trend to that of ACT participants. Indeed, it has been reported that anxiety is usually highest on the day of surgery, followed by the day before surgery and then the day after surgery (Goyal et al. 2025). However, the use of an experimental design allowed us to conclude that our intervention significantly fosters that trend. Furthermore, almost all participants in the ACT group were considered responders at the three follow‐ups, with a notable difference at 6 months (96% vs. 29% in TAU). Considering the associated ARR and NNT, ACT not only produced statistically significant improvements in pain interference but also did so with efficiency highly relevant to both clinical and service‐delivery perspectives.

The favourable clinical outcomes observed in the ACT group may also be partly attributable to high treatment adherence, with 84% of participants attending nearly all sessions. This is striking when compared with CLBP patients from the same hospital who received the same ACT protocol, among whom only 44% attended 7 or 8 sessions (Sanabria‐Mazo et al. 2023). Although the online program was implemented during the COVID‐19 pandemic, this difference may indicate particularly strong motivation among patients awaiting surgery to acquire psychological tools to cope with surgery‐related and pain‐related psychological distress. This interpretation is consistent with Salzmann et al. (2021), who found that a large proportion of more than 1000 patients scheduled for elective surgery reported a strong desire for preoperative psychological support.

Finally, we found no statistical evidence across any assessment time points that changes in psychological flexibility or pain acceptance explained improvements in clinical outcomes. This result was unexpected to us, considering the previous literature. Within ACT, these processes are considered key processes of change, as the primary aim is to reduce the impact of pain on a person's life by increasing psychological flexibility (Stockton et al. 2019). Individuals with high psychological flexibility tend to ‘accept’ pain, in the sense of continuing to pursue what matters to them while integrating it into their ongoing experience (Feliu‐Soler et al. 2018). In line with this, a small but growing body of ACT‐based mediation studies for chronic pain has identified indirect effects of acceptance on improvements in physical functioning from baseline to 6‐month follow‐up (Cederberg et al. 2016). However, the absence of significant indirect effects should be interpreted with caution. A possible explanation for the present results is that our sample was likely too small to provide sufficient statistical power to detect a mediated effect (Fritz and MacKinnon 2007).

These findings should be interpreted with several limitations in mind. First, there was no independent assessment of treatment fidelity or therapist competence, and all ACT sessions were delivered by a single therapist. Although this likely enhanced protocol consistency, it may limit the ability to draw conclusions about the intervention's reproducibility across providers and settings. As is typical in trials of psychological interventions, therapists and participants could not be blinded to treatment allocation. Second, adherence to between‐session home exercises was not systematically monitored, so the extent to which practice outside the sessions contributed to treatment gains remains unclear. Third, the trial was conducted at a single tertiary hospital with a limited sample size, which may inflate effect size estimates and reduce the precision and generalizability of the results. Moreover, the final analysed sample (mITT *n* = 54) was substantially smaller than the protocol‐specified target (*n* = 102), which may have limited statistical power, particularly for the mediation analyses, and increased the risk of Type II error. Fourth, all primary and secondary outcomes were based on self‐report questionnaires, and no objective indicators of functioning (e.g., performance‐based measures or return‐to‐work data) or systematic data on analgesic and opioid use were included, which would have strengthened the clinical interpretation of the findings. Finally, the mediation analyses were likely underpowered to detect small‐to‐moderate indirect effects, and we focused only on two core ACT processes (psychological flexibility and pain acceptance), potentially overlooking other relevant mechanisms (e.g., values‐based action).

Future research should replicate these findings in larger, multicenter trials with more diverse samples and longer follow‐up periods to evaluate the durability of ACT effects on postsurgical outcomes. Another aspect that remains unexplored is whether ACT has good value for money in this population. An economic evaluation (e.g., cost–utility and cost‐effectiveness analyses) embedded within a well‐powered RCT seems warranted. Including active control conditions (e.g., alternative psychological interventions or structurally equivalent education/support groups) would enable stronger tests of ACT's specificity. Further studies should also incorporate objective and clinically salient endpoints such as opioid consumption, time to functional recovery, return to work, and health economic outcomes, and a qualitative study to hear the voices of patients regarding the barriers and facilitators they have encountered, or the pros and cons of ACT when delivered before surgery.

## 5 Conclusions

This single‐blinded RCT provides evidence supporting the clinical relevance of a group‐based form of online ACT as an add‐on to usual care for the improvement of pain interference and depressive/anxiety symptoms, pain catastrophizing, pain‐related fear of movement, and pain‐related disability, both before surgery and through 6‐ and 12‐month post‐surgery. These improvements, along with reported adherence rates, suggest that perioperative psychological approaches may be a valuable strategy for supporting surgical candidates and improving their quality of life and surgical outcomes.

## Author Contributions

Juan R. Castaño‐Asins contributed to conceptualization, methodology, and writing – original draft. Juan P. Sanabria‐Mazo and Jaime Navarrete contributed to data curation, software, formal analysis, methodology, visualization, and writing – original draft. Juan V. Luciano, Víctor Pérez‐Solà, and Antonio Montes contributed to conceptualization, funding acquisition, investigation, project administration, supervision, and writing, review and editing. Luis M. Martín‐López, Gemma Vila‐Canet, Anna Isart‐Torruella, Alejandro Del Arco‐Churruca, Jesús Lafuente‐Baraza, Gemma Parramon‐Puig, and Francina Fonseca‐Casals contributed to writing, review and editing. All authors read and approved the final manuscript.

## Funding

Juan P. Sanabria‐Mazo holds a Juan de la Cierva postdoctoral contract awarded by the Spanish Ministry of Science (JDC2024‐053318‐I). Jaime Navarrete has a postdoctoral contract awarded by the Centre for Biomedical Research in Epidemiology and Public Health (CIBERESP; CB22/02/00052). The authors thank CIBERESP (CB22/02/00052) and CIBERSAM (CB/07/09/0010) for their support. The Institute of Health Carlos III (ISCIII) and CIBERESP did not play any role in the analysis and interpretation of data, in the writing of the manuscript, or in the decision to submit the article for publication.

## Disclosure

Generative artificial intelligence (AI) was not used in the preparation of this manuscript.

## Conflicts of Interest

Víctor Perez has received grants and served as a consultant, advisor, or CME speaker for the following entities: Almirall, AstraZeneca, Eli Lilly, GlaxoSmithKline, Ferrer, Johnson & Johnson, Lundbeck, Merck, Otsuka, Pfizer, Servier, Abye Mectronic, and the Spanish Ministry of Science and Innovation. The remaining authors declare no conflicts of interest.

## Data Availability

The data that support the findings of this study are available from the corresponding author upon reasonable request.
